# Alternations and Applications of the Structural and Functional Connectome in Gliomas: A Mini-Review

**DOI:** 10.3389/fnins.2022.856808

**Published:** 2022-04-11

**Authors:** Ziyan Chen, Ningrong Ye, Chubei Teng, Xuejun Li

**Affiliations:** ^1^Department of Neurosurgery, Xiangya Hospital, Central South University, Hunan, China; ^2^Hunan International Scientific and Technological Cooperation Base of Brain Tumor Research, Xiangya Hospital, Central South University, Changsha, China; ^3^Department of Neurosurgery, The First Affiliated Hospital, University of South China, Hengyang, China

**Keywords:** glioma, diffusion magnetic resonance imaging, functional magnetic resonance imaging, connectome, brain network

## Abstract

In the central nervous system, gliomas are the most common, but complex primary tumors. Genome-based molecular and clinical studies have revealed different classifications and subtypes of gliomas. Neuroradiological approaches have non-invasively provided a macroscopic view for surgical resection and therapeutic effects. The connectome is a structural map of a physical object, the brain, which raises issues of spatial scale and definition, and it is calculated through diffusion magnetic resonance imaging (MRI) and functional MRI. In this study, we reviewed the basic principles and attributes of the structural and functional connectome, followed by the alternations of connectomes and their influences on glioma. To extend the applications of connectome, we demonstrated that a series of multi-center projects still need to be conducted to systemically investigate the connectome and the structural–functional coupling of glioma. Additionally, the brain–computer interface based on accurate connectome could provide more precise structural and functional data, which are significant for surgery and postoperative recovery. Besides, integrating the data from different sources, including connectome and other omics information, and their processing with artificial intelligence, together with validated biological and clinical findings will be significant for the development of a personalized surgical strategy.

## Introduction

In the central nervous system (CNS), gliomas are the most common, but complex primary tumors. An average annual incidence rate of glioma is 6.7 per 100,000 individuals in China (Jiang et al., 2016; Zhao et al., 2021), which is similar to that in the United States (Ostrom et al., 2020). Genome-based molecular and clinical studies have revealed different classifications and subtypes of gliomas. Cancer-related molecular characteristics could be used to indicate the type of treatment modality, such as surgery and adjuvant therapies, as well as clinical outcomes. The 2021 World Health Organization (WHO) classification of CNS tumors (Wen and Packer, 2021) divided gliomas into 6 different types, including adult-type diffuse gliomas, pediatric-type diffuse low-grade gliomas (LGG), pediatric-type diffuse high-grade gliomas, circumscribed astrocytic gliomas, glioneuronal and neuronal tumors, and ependymomas. The classification and grading of gliomas combine clinical, pathological, histological, and especially molecular features. Molecular profiles, such as isocitrate dehydrogenase (IDH)-mutant, 1p19q co-deletion, the X-linked alpha-thalassemia mental retardation (ATRX)-mutant, Telomerase reverse transcriptase (TERT) promoter mutant, and O6-methylguanine–DNA methyltransferase (MGMT) promoter methylation, provide a microscopic view for predicting tumor biological behavior and patients’ outcomes. Besides, neuroradiological approaches non-invasively provide a macroscopic view for surgical resection and improving therapeutic effects.

In clinical practice, computed tomography (CT), magnetic resonance imaging (MRI), biphasic 2-deoxy-2-[18 F]fluoro-D-glucose positron emission tomography/CT (^18^F-FDG PET/CT), and neuronavigation are the main imaging methods to assess cancer metabolism, cancer progression, conditions of nerve fibers, and cortical and subcortical functions.

Along with the development of artificial intelligence (AI), quantitative data from CT and ordinary MR images, such as T1, T2, and T1-contrast-enhanced, and fluid-attenuated inversion recovery (FLAIR) images could be extracted and processed by machine learning or deep learning algorithms (Lambin et al., 2012; Chang et al., 2019). State-of-the-art neural networks could select shape, texture, and gray level of tumors for predicting tumor classification (Lee et al., 2020; Chakrabarty et al., 2021), segmentation (Tang et al., 2020; Rudie et al., 2021), grading (Naser and Deen, 2020), and even molecular parameters (Bangalore Yogananda et al., 2020; Choi et al., 2021).

Studies (Lundervold and Lundervold, 2019; Tomaszewski and Gillies, 2021) have investigated the imaging features, as well as their potential relationships with pathological and molecular characteristics. However, in clinical work, as some special gliomas are located in some functional areas or important subcortical areas, their physical properties with surrounding brain tissues and functional effects on both hemispheres are worthy of further investigation. Thus, special imaging sequences, including structural and functional MRI pulse sequences, can be exploited to determine the functional connectivity of tumors and other tissues, which are called brain tumor connectome.

The brain’s anatomical connectivity, or connectome, was defined as the map of neural connections in the brain (Hagmann, 2005). The connections emphasized the fact that the huge brain neuronal communication capacity and computational power critically relied on subtle and incredibly complex connectivity architecture through diffusion MRI (dMRI) (Mori and Barker, 1999; Sporns et al., 2005; Assaf and Pasternak, 2008), which was designed to assess the trajectory of protons in white matter fiber bundles, illustrating structural connectivity in the brain. Apart from dMRI, functional MRI (fMRI) (Ogawa et al., 1992; Logothetis, 2008) indirectly reflects the activity of electrical signals of neurons in a certain brain area by detecting changes in blood oxygen levels, thereby reflecting the functional connections of neurons. By analyzing the functional connections through task-fMRI or resting-state fMRI (rs-fMRI), we could construct the functional connectivity atlas, followed by establishment of the brain connectome that is composed of functional and structural connectivity. In general, as a key component of brain multi-omics, connectome, depending on improvement of imaging hardware and software (Wang et al., 2013), could be used to explore neuropsychiatric activities and their changes (Sporns, 2011). In the present study, we reviewed the structural–functional connectome attributes in normal human brains and their abnormalities and instabilities in glioma, and highlighted their potential applications in glioma clinical management.

## Structural Connectome and the Alternations in Gliomas

### Structural Connectome

**FIGURE 1:**
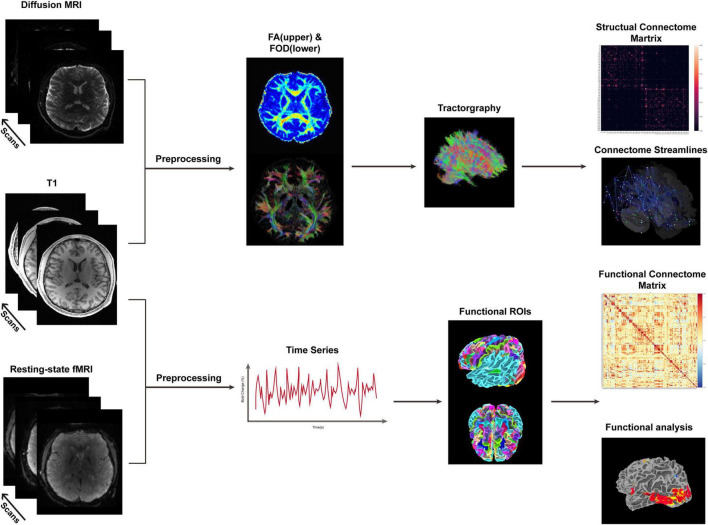
Workflow of diffusion MRI (dMRI) and resting-state functional MRI (fMRI). After image acquisition, images obtained by dMRI and fMRI were initially preprocessed by denoise, bias correction, motion correction, alignment, etc. Then, in dMRI, fiber orientation distribution (FOD), and fractional anisotropy (FA) were calculated for tractography, followed by structural connectome evaluation. In fMRI, time series sequences were used to assess the brain functional activities. Besides, seed-based approaches were used for the analysis of regions of interests (ROIs) and the graph theory was employed for the whole-brain functional connectome analysis.

According to the different degrees of free-water molecules in neurons, dMRI could distinguish the white matter (WM), gray matter (GM), and cerebrospinal fluid (CSF) by imposing gradients of magnetic fields and controlling the time intervals through different *b*-values (Cohen and Assaf, 2002). The myelinated axon of a neuron in one region of the brain extends to another region following a particular anatomic course or trajectory. Thus, the graph theory (Euler, 1741; Semmel et al., 2021), proposed in the 18th century, could provide a robust mathematical and practical foundation for structural connectome. Specifically, after acquiring ordinary MRI-T1, -T2, and diffusion sequences, as well as preprocessing the images including space alignment, image denoise, and field correction in a standard workflow, we could estimate the response functions of WM, GM, and CSF. Then, the response functions were applied to deconvolve the orientations of fibers and construct the tractography by various atlases (Tzourio-Mazoyer et al., 2002; Desikan et al., 2006; Fan et al., 2016; Glasser et al., 2016). A brain structural network graph would be architected by measuring the strengths and directions of neurons (Figure 1). Numerous studies (Hagmann, 2005; He et al., 2007; Toga et al., 2012; Liao et al., 2017) analyzed the dMRI tractography and network topology of the brain and revealed the long-range axonal network and the hierarchical organization of the functional inter-cortical connectivity. A small-world network architecture (Watts and Strogatz, 1998) has mainly demonstrated a low-cost, but high clustered network organization. Hub neuronal nodes as the centralities in the network could be connected together to enable an efficient and robust signaling in the network. A rich club is defined as a set of high-degree nodes that is more densely interconnected than predicted on the basis of the node degrees alone (de Reus and van den Heuvel, 2013), suggesting the theoretical basement of anatomical organization for data transmission. It is also a conservative network architecture across various species (Watts and Strogatz, 1998; Oh et al., 2014; Beul et al., 2015) and spatial scales (Das, 2020). Previous studies (Collin and van den Heuvel, 2013; van den Heuvel et al., 2015) demonstrated that seeking the differences of brain connectome between preterm and term infant brains is a vital step for understanding the developmental impairment caused by preterm.

Tractography constructed by high-resolution MRI systems provides the WM connections of cortico-cortical and cortico-subcortical spatial regions and their anatomic relationships with normal functions of awareness, cognition, behaviors, etc. Thus, abnormalities in structural connectome affected by tumors could reflect the biological behaviors, such as tumor progression and invasion.

### Structural Connectome Alternations in Gliomas

In gliomas, a number of scholars (Venkataramani et al., 2019, 2021; Venkatesh et al., 2019) have reported a relevant direct synaptic communication and electrical integration between tumors cells and neurons, providing the potential explanations of tumors, involving the neuronal signaling pathways. Glutamatergic synapses could medicate connections between presynaptic neurons and postsynaptic glioma cells and form a positive current, promoting calcium-related invasiveness of tumor cells and glioma proliferation until it would be inhibited by glutamate receptor antagonists (Venkataramani et al., 2019). Meanwhile, glioma interconnections *via* gap junctions (Osswald et al., 2015) and membrane tubes (Jung et al., 2017; Weil et al., 2017) could construct a self-repairing and resistant network, disturbing the normal brain network, as well as promoting tumor progression and therapeutic resistance.

Therefore, implementation of dMRI and establishment of tractography might detect the accumulation of the abnormal cells. As we mentioned above, hub neuronal regions play a central role in the brain small-world network and can be clustered into a rich club. Glioma patients, however, have more hub-related connections in the contralateral hemisphere (Yu et al., 2016; Douw et al., 2019; Yuan T. et al., 2020), while a slight difference in the ipsilateral hemisphere was found. These contralesional connections suggested the decompensation of structure and function and the particular redistribution of connectivity (Crossley et al., 2014; Stam, 2014). The cascade network failure theory (Buldyrev et al., 2010; van den Heuvel and Sporns, 2019), indicating the overloading of functional hubs and the increase of connectivity, has been studied in Alzheimer’s disease (Jones et al., 2016) and schizophrenia (van den Heuvel and Sporns, 2019). More specifically, if a hub bears the throughput beyond its threshold, it could lead to catastrophic system collapse. For the whole brain level, network analysis showed decreased global and local connectivity efficiency in the ipsilesional hemisphere (Fekonja et al., 2021); Jutten et al. (2021) investigated fractional anisotropy (FA) of dMRI and achieved similar results of structural connectivity and systemic microstructural WM dis-integrity, especially in the IDH-wild-type (IDH-wt) group due to the worse biological attributes (Eckel-Passow et al., 2015). In addition, patients with IDH-wt and a greater lesion volume have significantly lower connectivity efficiency and cognitive capability (Kesler et al., 2017). In other words, functional connectivity was traditionally considered based on brain anatomy and the structural connectivity. Functional aberrations in cognition or perception might be caused by impairment of structural integrity. Wei et al. (2021) quantified the global and regional connectome disruptions in individual glioblastoma patients and investigated the prognostic value of connectome disruptions and topological properties using a structural connectome approach based on diffusion MRI, and they demonstrated that glioblastoma patients had decreased segregation and increased integration, which could potentially provide a useful biomarker for patient stratification.

Not only has structural connectivity provided the orientations of neurons for formulating surgical plan and navigation (Abdullah et al., 2013; Henderson et al., 2020), but it also could be advantageous for prediction of molecular characteristics and prognosis evaluation. On the one hand, determining the infiltration or displacement of cortical mapping and WM tracts would be significant for the extent of tumor removal and normal brain tissue protection (Castellano et al., 2012; Abhinav et al., 2015). Gliomas are the most common intra-axial brain tumors characterized by invasion into the surrounding WM tracts; thus, the combination of tractography with the operative navigation could show the adjacency between bulks and edemas of tumors and WM skeletons. Different approaches have been presented for mapping brain connectivity, and defining nodes and edges at each scale, so as to protect the integrity of the brain network. On the other hand, numerous studies have reported that gliomas have specific molecular parameters, such as IDH-mutation (Chen et al., 2017; Kesler et al., 2019) and MGMT promoter methylation (Chen et al., 2017). To date, deep neural networks have been utilized to extract dMRI metrics like ADC and FA value, and to assist IDH, MGMT, and other molecule subtype classification and prognosis prediction of glioma patients (Aliotta et al., 2019; Z. Huang et al., 2021; Yan et al., 2021).

## Functional Connectome and the Alternations in Gliomas

### Functional Connectome

Oriented in 1990s, fMRI measuring hemodynamic changes after enhanced neuronal activity has been applied on neuroscience research and clinical practice (Ogawa and Lee, 1990; Logothetis, 2008). When one stays awake and produces thinking activities, his/her related brain area would produce electrophysiological signals, which could be detected indirectly by changes in the oxyhemoglobin (HbO_2_) and deoxyhemoglobin (Logothetis and Wandell, 2004). Spatiotemporal properties of blood oxygenation level-dependent (BOLD) fMRI showed higher intensity signals when electrical activities happened due to higher HbO_2_ levels. Although the imbalance of supply and consumption of HbO_2_ remained elusive (Drew, 2019), one theory indicated that dilation of arteries increases neuronal activities, so as to produce higher HbO_2_ levels (Drew et al., 2011). Previous studies have proposed several models (Malonek and Grinvald, 1996) to simulate the relationships between BOLD signals and hemodynamic response function (HRF) (Cohen, 1997; Friston et al., 1998) and revealed linear relationships. Similar to the dMRI processing pipeline, fMRI data were initially preprocessed and HRF was then applied to deconvolute and fit the time series signals by linear regressive models. Recently, surface-based processing (Fischl et al., 1999) has become a popular approach to overcome the calculation and atlas problems, increasing the specificity of cortical activation patterns (Oosterhof et al., 2011; Saad and Reynolds, 2012; Brodoehl et al., 2020). Graph theory and other methods [independent component analysis (ICA), seed-based analysis, etc.], which are compatible to dMRI analysis theories, were also implemented according to the experiment design. Finally, a brain functional connectome was established to evaluate regional brain activity and functional connectivity (Figure 1).

To better evaluate a particular function parcellation in the brain area, task-fMRI is typically characterized more precisely to delineate the relationships between tasks and electrophysiological responses. Different cognitive tasks, including executive functioning, emotional processing, language, thinking, and implicit learning tasks, could be implemented by block, event-related, or mixed experiment design (Mwansisya et al., 2017). In clinical practice, however, doing task-fMRI needs more complex stimulation and recording systems, which might be difficult for tolerance of tumor patients. Rs-fMRI has also concentrated on brain functional mapping with subjects staying awake, while not performing any task (van den Heuvel and Hulshoff Pol, 2010). Studies have reported the resting-state networks, describing functional connectivity of brain regions in the resting state. The networks include the motor network, the visual and auditory network, dorsal and ventral attention network, central executive network (CEN), default mode network (DMN), and salience network (SN) (Spreng et al., 2013; Raichle, 2015; Yeshurun et al., 2021). Among these networks, CEN, DMN, and SN are the core neurocognitive-related networks. CEN, also known as frontoparietal network, contains dorsolateral prefrontal cortex and posterior parietal cortex, and is widely co-activated in cognitive behaviors and is involved in working memory (Menon, 2011). DMN, including the posterior medial cortex, medial prefrontal cortex, and temporoparietal junction, has shown a higher connectivity during resting state, and it was recognized as an active and dynamic intrinsic system to integrate information (Yeshurun et al., 2021). SN refers to cortical hubs in the dorsal anterior cingulate cortex, insular cortex, temporal pole, and amygdala, and it is related to links between the stimulus from external environment and the response of other networks dynamically (Seeley, 2019). A triple network model, consisting of CEN, DMN, and SN, has been proposed to understand the cross-network interactions and human brain state transition (Menon, 2011). Studies have reported that these non-traditional networks play key roles in the human brain in different diseases including Alzheimer’s disease and mental disorders (Menon, 2011; Liston et al., 2014; Satpute and Lindquist, 2019; Misiura et al., 2020; Martin-Subero et al., 2021).

Task-fMRI and rs-fMRI provide a non-invasive method to evaluate the active regions in the brain and their relationships. The spatial and temporal resolution, as well as the difference of HRF, limit the fMRI applications in causal inference; however, it is reliable to measure the fMRI for hypothesis testing (Olszowy et al., 2019). Recently, the molecular mechanism of functional connectivity was investigated by the correlation analysis between RNA-sequence and functional connectivity strength of rs-fMRI (Zhu et al., 2021), providing a potential to explore the genetic nature of the functional connectome.

### Functional Connectome Alternations in Gliomas

Functional connectivity in gliomas was altered by the aggressiveness of glioma cells and the increased oxygenated blood flow in tumor vasculature (Warmuth et al., 2003; Hadjiabadi et al., 2018). Incorporating the assessment of the rs-fMRI network into the traditional glioma mapping approaches could be a versatile method for anatomical and functional localization and evaluation of molecular parameters (Ghinda et al., 2018; Stoecklein et al., 2020). Prior studies have reported a higher mean connectivity (Otten et al., 2012) and brain-wide disrupted networks associated with tumor-related remodeling of the neurovasculature (Hadjiabadi et al., 2018). In particular, several research groups measured the DMN in glioma by rs-fMRI and observed the increased integration of DMN in hippocampal areas, but the decreased integration in prefrontal and posterior cingulate cortex regions, and a trend of decreasing global connectivity with a higher WHO grade (Esposito et al., 2012; Harris et al., 2014; Maniar et al., 2021). The decreased DMN integration and deactivation may lead to cognitive decline of patients. SN connectivity in glioma could be reduced with an increased amplitude of low-frequency fluctuations (ALFFs), reflecting intrinsic brain activity, as well as promoting specific brain circuits to participate in cognitive tasks. The increased ALFF in the tumor contralateral regions may be explained by functional compensation due to neuronal plasticity and neuro-vascular uncoupling of tumor ipsilateral regions (Yang et al., 2021). Thus, tumors that are close to hubs of these networks could lead to dysfunction, but without obvious clinical manifestations due to a compensatory response of other areas. For traditional functional networks such as language and sensorimotor networks, studies through seed-based and ICA approaches revealed significantly reduced integrity and connectivity (Briganti et al., 2012; Niu et al., 2014; Mallela et al., 2016; Vassal et al., 2017; Yuan B. et al., 2020). Specifically, the reduction had a distinct pattern modulated by tumor position. The closer tumor is to hub regions, the more severe the effects to the function. These findings were consistent with the clinical manifestations in patients whose tumors were located in language, sensory, or motor regions.

Functional network also has the small-world attribute, and studies have reported the disturbed small-world manner and the decreased connective efficiency in LGG patients (Xu et al., 2013; Huang et al., 2014), who mainly suffered from cognitive deficits, memory reduction, and psychomotor dysfunction. However, an increased local efficiency and a higher local clustering coefficient were observed in the lesion areas, suggesting the potential preservation mechanism in small-world topology (Huang et al., 2014; Park et al., 2016). The specific biological behaviors of breaking and protecting small-world properties have not been fully explored, and a large number of studies have reported that IDH-wt was highly associated with the dysfunction of functional network (Derks et al., 2019; Jutten et al., 2020), indicating that infiltration and aggression of genetic characteristics of gliomas may affect the functional network. Recently, Mandal et al. (2020) established a model that combined cellular types and transcription of genetic drivers of glioma genesis with graph theory-based functional hub-based analysis, and found patterns of glioma locations. Their model explained over half of the variance in glioma location frequency, brain regions populated with putative cells of origin for glioma, neural stem cells, and oligodendrocyte precursor cells. On the other hand, the study also explained that the occupation and destruction of brain functional hubs by glioma decreased global small-world clustering. The complexity of biological characteristics of glioma and distribution of their locations are still accompanied by some problems in functional connectomes, indicating the necessity to investigate the correlations between tumor biology and brain function.

Mapping the functional connectome also provides sufficient data for surgical planning and prognosis assessment. In clinical practice, awake mapping using direct electrical stimulation (DES) of the brain can be achieved in surgery for gliomas in functional regions (Duffau, 2015). Anatomo-functional location by dMRI and rs-MRI before surgery could assist DES for precise functional protection. Qiu et al. (2017) demonstrated that using intraoperative rs-fMRI could directly localize the functional areas intraoperatively and avoid the risk of intraoperative seizures due to direct cortical stimulation. Postoperatively, patients mainly prefer to receive radiotherapy and chemotherapy. Thus, functional connectome analysis could evaluate the effects of treatment, brain recovery, and quality of life in patients. However, conflicts still exist between the tumor resection and brain network protection, and these traditional and non-traditional networks were invaded or pushed by tumor bulks, resulting in different operative approaches and tumor resection ranges. In future research, the balance between connectome protection and surgery and radio-chemotherapy will be deeply investigated.

## Discussion

In 2009, the National Institutes of Health proposed the Human Connectome Project (HCP) with a primary goal of delineating the typical patterns of structural and functional connectivity in the brains of healthy individuals and patients with different psychiatric and neurologic diseases (Van Essen et al., 2012, 2013; Barch et al., 2013). To date, diseases, such as Alzheimer’s disease (Daianu et al., 2015; Sun et al., 2020), epilepsy (Hwang et al., 2020), and mental disorders (Korgaonkar et al., 2020), have been profoundly assessed in alternations of connectome. Glioma-related connectome projects have not been presented, although a large number of studies have illustrated the reduction in structural and functional connectivity as we mentioned. From traditional views, we paid further attention to tumor resection and identification of molecular characteristics. A series of pertinent brain macro perspectives and multi-center projects still need to be conducted to extend the knowledge of glioma, although connections between structural and functional connectome have remained obscure due to limitations of MRI technology. For example, in clinical practice, dMRI and fMRI are not always acquired due to the financial status of patients and or scanner machine-time shortage especially in developing countries. On the other hand, differences in sequence parameters would also make it difficult to achieve unbiased data standardization. From the patients’ view, many patients with glioma have alternations in cognition, motion, and attention and high risks of secondary epilepsy, so they may not cooperate with physicians with regard to scanning for a long time (Silva et al., 2018). Technologically, dMRI and fMRI techniques are limited by the resolutions. Lower signal-to-noise ratio (SNR) and resolutions may reflect poor image qualities (Jha et al., 2020; Morales, 2021; Turesky et al., 2021). In particular, for dMRI, the partial volume effect and the disability for non-Gaussian diffusion (Assaf and Pasternak, 2008) are the main difficulties that lead to inaccurate fiber tracking. For fMRI, excessive blood supply in glioma could uncouple the BOLD signal and susceptibility motion artifact remains an important concern for task design and imaging time (Silva et al., 2018; Morales, 2021). Besides, structural–functional coupling in glioma will be explored deeply in the future. Structural–functional coupling describes anatomical support for functional communications across brain regions (Baum et al., 2020; Gu et al., 2021). Zhang et al. (2021) reported the structural–functional connectome of language in glioma-induced aphasia, and patients with aphasia were without sufficient functional compensation in the supplementary motor area, which was mainly involved in aphasia. Traditionally, correlation analysis was commonly used for the structural connection (SC) and functional connection (FC) strength to obtain a structural–functional coupling matrix. However, this method still has the disadvantage of FC automatically forming stronger in the absence of a direct structural link (Honey et al., 2009); Sarwar et al. (2021) trained a deep learning network to predict the FC based on SC as input signals, and correlations were computed between pairs of subjects or between distinct modalities within the same individual to assess the structural–functional coupling strength. Hybrid connectivity ICA, which could extract the connectivity traits from SC and FC, was also applied to explore structural–functional coupling patterns (Amico and Goni, 2018). To improve the image qualities, 7-T or higher field MRI would be significant for providing higher-resolution and lower-noise images, containing more precise information for brain’s structure and function. At the same time, generative adversarial networks were used to generate and standardize the MRI data from different machines and to improve the image resolutions; thus, researchers can share and use the data stored in the cloud and perform large-scale clinical studies (Kazuhiro et al., 2018).

With the improvement of the brain–computer interface (BCI) that uses algorithms to decode brain signals, non-invasive brain signals, which could be obtained by converging multimodal MRI data to electrophysiological signals, could elevate the accuracy and efficiency of decoding. During the clinical management of glioma, aphasia or motion disability may occur due to tumor invasion or surgery. At present, electroencephalography (EEG)-based BCI has been widely used to decode brain signals *via* encode–decode deep learning models for patients with language or motor dysfunction (Makin et al., 2020; Li et al., 2021). Structural and functional connectome combined with EEG will provide more sufficient and precise data, which are beneficial for postoperative recovery and protection. Besides, BCI can improve cancer patients’ quality of life.

**FIGURE 2:**
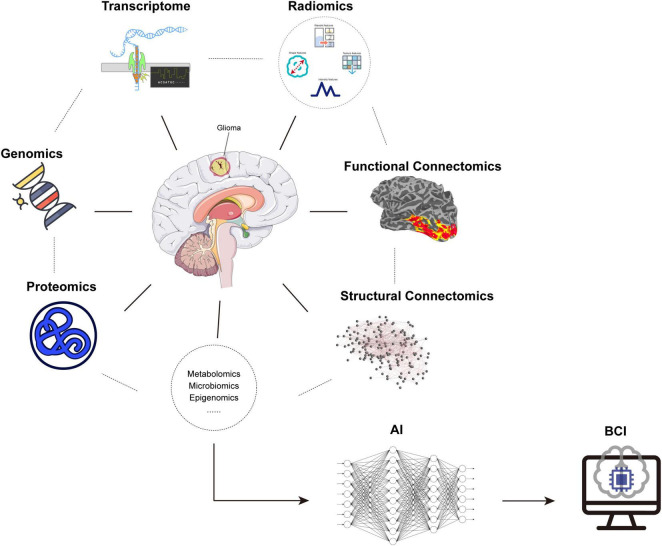
Glioma-related multi-omics. Combination of omics including genomics, transcriptome, proteomics, radiomics, connectomics, etc. from microscopy to macroscopy and application of AI provided a novel and detailed approach for glioma, accompanied by a potential for BCI. AI: artificial intelligence, BCI: brain–computer interface.

The meta-network analysis has been proposed, underlying the uniquely human propensity, to learn complex abilities and explain how post-lesional reshaping can lead to some degrees of functional compensation in patients with brain injury. Herbet and Duffau (2020) reviewed the progress in functional anatomy of the human brain and defined a meta-network that could integrate high-level cerebral functions from dynamic spatiotemporal dimensions, so as to provide structural and functional reshaping. Different from the existing approaches, the meta-network considers the systematic interactions rather than special brain regions, and it is more robust to noise interference. Adaptive changes of large-scale connectivity, causing cognition, perception, and emotion, were analyzed with the meta-network to better understand the underlying and constant connection patterns and their stability. Duffau (2021) also concentrated on applying the meta-network to glioma topography and surgery. For better tumor resection and brain protection, the interactive loop between tumor invasion, brain functional compensation, and the therapeutic strategy should be established. When the tumor cells involve the critical pathways, development of dynamic multimodal imaging methods may facilitate disconnecting the tumor-neural signaling and preserve the normal connectome. In order to treat glioma more effectively, multiple views and analysis approaches must correspond from molecular parameters to fiber orientations to global brain functional connectivity. However, for MRI data analysis, Bowring et al. (2019) explored the different tools with the same data and pipeline in fMRI and found inconsistent results. The results concluded that the effort would be strengthened for analysis scripts and statistical models. To enable the confirmation of these tools, deep learning algorithms could help to analyze the scripts of different tools, and construct more robust statistical models. Apart from dMRI and fMRI data themselves, multi-omics analysis provides a novel and detailed approach to glioma (Figure 2). Recent studies have investigated using the multi-omics sources including genomics, transcriptome, and proteomics for the glioma classification, prognosis prediction, and identification of therapeutic targets *via* machine learning in open datasets (Kamoun et al., 2016; Biswas and Chakrabarti, 2020; Zeng et al., 2021). Our studies have used radiomics in MRI to predict the key molecular status of glioma by deep learning networks (Chang et al., 2018; Zhang et al., 2020). However, there are still lack of systematic studies to combine the connectome and other scales of information of glioma. Thus, integrating data from different sources, and their processing with AI, together with validated biological and clinical findings will be significant for the development of a personalized surgical strategy.

In conclusion, from a personality treatment perspective, to reliably diagnose and manage gliomas, high-field imaging and data processing algorithms enable the achievement of high-resolution images and performing real-time analysis of multimodal MRI data, so as to construct the structural–functional connectome. Additionally, further systemic and multi-omics studies are required to fully understand the mechanisms of alternations in gliomas. In addition to the changes in connectome, the biological and molecular characteristics should be explored. Thus, additional efforts need to be dedicated to illustrate the nature of gliomas and to develop more effective treatments for gliomas.

## Author Contributions

XL designed the study. ZC performed the research and wrote the manuscript. NY and CT helped to revise the manuscript. All authors have made a substantial, direct, and intellectual contribution to the work, and approved it for publication.

## Conflict of Interest

The authors declare that the research was conducted in the absence of any commercial or financial relationships that could be construed as a potential conflict of interest.

## Publisher’s Note

All claims expressed in this article are solely those of the authors and do not necessarily represent those of their affiliated organizations, or those of the publisher, the editors and the reviewers. Any product that may be evaluated in this article, or claim that may be made by its manufacturer, is not guaranteed or endorsed by the publisher.

## References

[B1] Abdullah K. G. Lubelski D. Nucifora P. G. Brem S. (2013). Use of diffusion tensor imaging in glioma resection. *Neurosurg. Focus* 34:E1. 10.3171/2013.1.FOCUS12412 23544405

[B2] Abhinav K. Yeh F. C. Mansouri A. Zadeh G. Fernandez-Miranda J. C. (2015). High-definition fiber tractography for the evaluation of perilesional white matter tracts in high-grade glioma surgery. *Neuro Oncol.* 17 1199–1209. 10.1093/neuonc/nov113 26117712 PMC4588761

[B3] Aliotta E. Nourzadeh H. Batchala P. P. Schiff D. Lopes M. B. Druzgal J. T. (2019). Molecular Subtype Classification in Lower-Grade Glioma with Accelerated DTI. *AJNR Am. J. Neuroradiol.* 40 1458–1463. 10.3174/ajnr.A6162 31413006 PMC7048441

[B4] Amico E. Goni J. (2018). Mapping hybrid functional-structural connectivity traits in the human connectome. *Netw. Neurosci.* 2 306–322. 10.1162/netn_a_00049 30259007 PMC6145853

[B5] Assaf Y. Pasternak O. (2008). Diffusion tensor imaging (DTI)-based white matter mapping in brain research: a review. *J. Mol. Neurosci.* 34 51–61. 10.1007/s12031-007-0029-0 18157658

[B6] Bangalore Yogananda C. G. Shah B. R. Vejdani-Jahromi M. Nalawade S. S. Murugesan G. K. Yu F. F. (2020). A novel fully automated MRI-based deep-learning method for classification of IDH mutation status in brain gliomas. *Neuro Oncol.* 22 402–411. 10.1093/neuonc/noz199 31637430 PMC7442388

[B7] Barch D. M. Burgess G. C. Harms M. P. Petersen S. E. Schlaggar B. L. Corbetta M. (2013). Function in the human connectome: task-fMRI and individual differences in behavior. *Neuroimage* 80 169–189. 10.1016/j.neuroimage.2013.05.033 23684877 PMC4011498

[B8] Baum G. L. Cui Z. Roalf D. R. Ciric R. Betzel R. F. Larsen B. (2020). Development of structure-function coupling in human brain networks during youth. *Proc. Natl. Acad. Sci. U. S. A.* 117 771–778. 10.1073/pnas.1912034117 31874926 PMC6955327

[B9] Beul S. F. Grant S. Hilgetag C. C. (2015). A predictive model of the cat cortical connectome based on cytoarchitecture and distance. *Brain Struct. Funct.* 220 3167–3184. 10.1007/s00429-014-0849-y 25062666 PMC4575693

[B10] Biswas N. Chakrabarti S. (2020). Artificial Intelligence (AI)-Based Systems Biology Approaches in Multi-Omics Data Analysis of Cancer. *Front. Oncol.* 10:588221. 10.3389/fonc.2020.588221 33154949 PMC7591760

[B11] Bowring A. Maumet C. Nichols T. E. (2019). Exploring the impact of analysis software on task fMRI results. *Hum. Brain Mapp.* 40 3362–3384. 10.1002/hbm.24603 31050106 PMC6618324

[B12] Briganti C. Sestieri C. Mattei P. A. Esposito R. Galzio R. J. Tartaro A. (2012). Reorganization of functional connectivity of the language network in patients with brain gliomas. *AJNR Am. J. Neuroradiol.* 33 1983–1990. 10.3174/ajnr.A3064 22555573 PMC7964610

[B13] Brodoehl S. Gaser C. Dahnke R. Witte O. W. Klingner C. M. (2020). Surface-based analysis increases the specificity of cortical activation patterns and connectivity results. *Sci. Rep.* 10:5737. 10.1038/s41598-020-62832-z 32235885 PMC7109138

[B14] Buldyrev S. V. Parshani R. Paul G. Stanley H. E. Havlin S. (2010). Catastrophic cascade of failures in interdependent networks. *Nature* 464 1025–1028. 10.1038/nature08932 20393559

[B15] Castellano A. Bello L. Michelozzi C. Gallucci M. Fava E. Iadanza A. (2012). Role of diffusion tensor magnetic resonance tractography in predicting the extent of resection in glioma surgery. *Neuro Oncol.* 14 192–202. 10.1093/neuonc/nor188 22015596 PMC3266379

[B16] Chakrabarty S. Sotiras A. Milchenko M. LaMontagne P. Hileman M. Marcus D. (2021). MRI-based Identification and Classification of Major Intracranial Tumor Types by Using a 3D Convolutional Neural Network: a Retrospective Multi-institutional Analysis. *Radiol. Artif. Intell.* 3:e200301. 10.1148/ryai.2021200301 34617029 PMC8489441

[B17] Chang K. Bai H. X. Zhou H. Su C. Bi W. L. Agbodza E. (2018). Residual Convolutional Neural Network for the Determination of IDH Status in Low- and High-Grade Gliomas from MR Imaging. *Clin. Cancer Res.* 24 1073–1081. 10.1158/1078-0432.CCR-17-2236 29167275 PMC6051535

[B18] Chang K. Beers A. L. Bai H. X. Brown J. M. Ly K. I. Li X. (2019). Automatic assessment of glioma burden: a deep learning algorithm for fully automated volumetric and bidimensional measurement. *Neuro Oncol.* 21 1412–1422. 10.1093/neuonc/noz106 31190077 PMC6827825

[B19] Chen L. Zhang H. Thung K. H. Liu L. Lu J. Wu J. (2017). Multi-label Inductive Matrix Completion for Joint MGMT and IDH1 Status Prediction for Glioma Patients. *Med. Image Comput. Comput. Assist. Interv.* 10434 450–458. 10.1007/978-3-319-66185-8_51 29770368 PMC5951635

[B20] Choi Y. S. Bae S. Chang J. H. Kang S. G. Kim S. H. Kim J. (2021). Fully automated hybrid approach to predict the IDH mutation status of gliomas via deep learning and radiomics. *Neuro Oncol.* 23 304–313. 10.1093/neuonc/noaa177 32706862 PMC7906063

[B21] Cohen M. S. (1997). Parametric analysis of fMRI data using linear systems methods. *Neuroimage* 6 93–103. 10.1006/nimg.1997.0278 9299383

[B22] Cohen Y. Assaf Y. (2002). High b-value q-space analyzed diffusion-weighted MRS and MRI in neuronal tissues - a technical review. *NMR Biomed.* 15 516–542. 10.1002/nbm.778 12489099

[B23] Collin G. van den Heuvel M. P. (2013). The ontogeny of the human connectome: development and dynamic changes of brain connectivity across the life span. *Neuroscientist* 19 616–628. 10.1177/1073858413503712 24047610

[B24] Crossley N. A. Mechelli A. Scott J. Carletti F. Fox P. T. McGuire P. (2014). The hubs of the human connectome are generally implicated in the anatomy of brain disorders. *Brain* 137 2382–2395. 10.1093/brain/awu132 25057133 PMC4107735

[B25] Daianu M. Jahanshad N. Nir T. M. Jack C. R. Jr. Weiner M. W. Bernstein M. A. (2015). Rich club analysis in the Alzheimer’s disease connectome reveals a relatively undisturbed structural core network. *Hum. Brain Mapp.* 36 3087–3103. 10.1002/hbm.22830 26037224 PMC4504816

[B26] Das A. B. (2020). Small-world networks of prognostic genes associated with lung adenocarcinoma development. *Genomics* 112 4078–4088. 10.1016/j.ygeno.2020.07.018 32659327

[B27] de Reus M. A. van den Heuvel M. P. (2013). Rich club organization and intermodule communication in the cat connectome. *J. Neurosci.* 33 12929–12939. 10.1523/JNEUROSCI.1448-13.2013 23926249 PMC6619725

[B28] Derks J. Kulik S. Wesseling P. Numan T. Hillebrand A. van Dellen E. (2019). Understanding cognitive functioning in glioma patients: the relevance of IDH-mutation status and functional connectivity. *Brain Behav.* 9:e01204. 10.1002/brb3.1204 30809977 PMC6456787

[B29] Desikan R. S. Segonne F. Fischl B. Quinn B. T. Dickerson B. C. Blacker D. (2006). An automated labeling system for subdividing the human cerebral cortex on MRI scans into gyral based regions of interest. *Neuroimage* 31 968–980. 10.1016/j.neuroimage.2006.01.021 16530430

[B30] Douw L. Miller J. J. Steenwijk Ma D Stufflebeam S. M. Gerstner E. R. (2019). Altered structural hub connectivity and its clinical relevance in glioma. *bioRxiv* [Preprint]. 10.1101/610618

[B31] Drew P. J. (2019). Vascular and neural basis of the BOLD signal. *Curr. Opin. Neurobiol.* 58 61–69. 10.1016/j.conb.2019.06.004 31336326 PMC6859204

[B32] Drew P. J. Shih A. Y. Kleinfeld D. (2011). Fluctuating and sensory-induced vasodynamics in rodent cortex extend arteriole capacity. *Proc. Natl. Acad. Sci. U. S. A.* 108 8473–8478. 10.1073/pnas.1100428108 21536897 PMC3100929

[B33] Duffau H. (2015). Awake mapping of the brain connectome in glioma surgery: concept is stronger than technology. *Eur. J. Surg. Oncol.* 41 1261–1263. 10.1016/j.ejso.2015.05.009 26095703

[B34] Duffau H. (2021). Brain connectomics applied to oncological neuroscience: from a traditional surgical strategy focusing on glioma topography to a meta-network approach. *Acta Neurochir.* 163 905–917. 10.1007/s00701-021-04752-z 33564906

[B35] Eckel-Passow J. E. Lachance D. H. Molinaro A. M. Walsh K. M. Decker P. A. Sicotte H. (2015). Glioma Groups Based on 1p/19q, IDH, and TERT Promoter Mutations in Tumors. *N. Engl. J. Med.* 372 2499–2508. 10.1056/NEJMoa1407279 26061753 PMC4489704

[B36] Esposito R. Mattei P. A. Briganti C. Romani G. L. Tartaro A. Caulo M. (2012). Modifications of default-mode network connectivity in patients with cerebral glioma. *PLoS One* 7:e40231. 10.1371/journal.pone.0040231 22808124 PMC3392269

[B37] Euler L. (1741). Solutio problematis ad geometriam situs pertinentis. *Comment. Acad. Sci. Petropol.* 8 128–140.

[B38] Fan L. Li H. Zhuo J. Zhang Y. Wang J. Chen L. (2016). The Human Brainnetome Atlas: a New Brain Atlas Based on Connectional Architecture. *Cereb. Cortex* 26 3508–3526. 10.1093/cercor/bhw157 27230218 PMC4961028

[B39] Fekonja L. S. Wang Z. Cacciola A. Roine T. Aydogan B. D. Mewes D. (2021). Network analysis shows decreased ipsilesional structural connectivity in glioma patients. *medRxiv* [Preprint]. 10.1101/2021.06.22.21259319 PMC8943189 35322812

[B40] Fischl B. Sereno M. I. Dale A. M. (1999). Cortical surface-based analysis. II: inflation, flattening, and a surface-based coordinate system. *Neuroimage* 9 195–207. 10.1006/nimg.1998.0396 9931269

[B41] Friston K. J. Fletcher P. Josephs O. Holmes A. Rugg M. D. Turner R. (1998). Event-related fMRI: characterizing differential responses. *Neuroimage* 7 30–40. 10.1006/nimg.1997.0306 9500830

[B42] Ghinda D. C. Wu J. S. Duncan N. W. Northoff G. (2018). How much is enough-Can resting state fMRI provide a demarcation for neurosurgical resection in glioma? *Neurosci. Biobehav. Rev.* 84 245–261. 10.1016/j.neubiorev.2017.11.019 29198588

[B43] Glasser M. F. Coalson T. S. Robinson E. C. Hacker C. D. Harwell J. Yacoub E. (2016). A multi-modal parcellation of human cerebral cortex. *Nature* 536 171–178. 10.1038/nature18933 27437579 PMC4990127

[B44] Gu Z. Jamison K. W. Sabuncu M. R. Kuceyeski A. (2021). Heritability and interindividual variability of regional structure-function coupling. *Nat. Commun.* 12:4894. 10.1038/s41467-021-25184-4 34385454 PMC8361191

[B45] Hadjiabadi D. H. Pung L. Zhang J. Ward B. D. Lim W. T. Kalavar M. (2018). Brain tumors disrupt the resting-state connectome. *Neuroimage Clin.* 18 279–289. 10.1016/j.nicl.2018.01.026 29876248 PMC5987800

[B46] Hagmann P. (2005). *From diffusion MRI to brain connectomics.* Lausanne: EPFL.

[B47] Harris R. J. Bookheimer S. Y. Cloughesy T. F. Kim H. J. Pope W. B. Lai A. (2014). Altered functional connectivity of the default mode network in diffuse gliomas measured with pseudo-resting state fMRI. *J. Neurooncol.* 116 373–379. 10.1007/s11060-013-1304-2 24234804 PMC6763342

[B48] He Y. Chen Z. J. Evans A. C. (2007). Small-world anatomical networks in the human brain revealed by cortical thickness from MRI. *Cereb. Cortex* 17 2407–2419. 10.1093/cercor/bhl149 17204824

[B49] Henderson F. Abdullah K. G. Verma R. Brem S. (2020). Tractography and the connectome in neurosurgical treatment of gliomas: the premise, the progress, and the potential. *Neurosurg. Focus* 48:E6. 10.3171/2019.11.FOCUS19785 32006950 PMC7831974

[B50] Herbet G. Duffau H. (2020). Revisiting the Functional Anatomy of the Human Brain: toward a Meta-Networking Theory of Cerebral Functions. *Physiol. Rev.* 100 1181–1228. 10.1152/physrev.00033.2019 32078778

[B51] Honey C. J. Sporns O. Cammoun L. Gigandet X. Thiran J. P. Meuli R. (2009). Predicting human resting-state functional connectivity from structural connectivity. *Proc. Natl. Acad. Sci. U. S. A.* 106 2035–2040. 10.1073/pnas.0811168106 19188601 PMC2634800

[B52] Huang Q. Zhang R. Hu X. Ding S. Qian J. Lei T. (2014). Disturbed small-world networks and neurocognitive function in frontal lobe low-grade glioma patients. *PLoS One* 9:e94095. 10.1371/journal.pone.0094095 24714669 PMC3979755

[B53] Huang Z. Lu C. Li G. Li Z. Sun S. Zhang Y. (2021). Prediction of Lower Grade Insular Glioma Molecular Pathology Using Diffusion Tensor Imaging Metric-Based Histogram Parameters. *Front. Oncol.* 11:627202. 10.3389/fonc.2021.627202 33777772 PMC7988075

[B54] Hwang G. Hermann B. Nair V. A. Conant L. L. Dabbs K. Mathis J. (2020). Brain aging in temporal lobe epilepsy: chronological, structural, and functional. *Neuroimage Clin.* 25:102183. 10.1016/j.nicl.2020.102183 32058319 PMC7016276

[B55] Jha R. R. Jaswal G. Nigam A. Bhavsar A. (2020). “Advances and challenges in fMRI and DTI techniques,” in *Intelligent Data Security Solutions for e-Health Applications*, eds Singh A. K. Elhoseny M. (Amsterdam: Elsevier).

[B56] Jiang T. Mao Y. Ma W. Mao Q. You Y. Yang X. (2016). CGCG clinical practice guidelines for the management of adult diffuse gliomas. *Cancer Lett.* 375 263–273. 10.1016/j.canlet.2016.01.024 26966000

[B57] Jones D. T. Knopman D. S. Gunter J. L. Graff-Radford J. Vemuri P. Boeve B. F. (2016). “Cascading network failure across the Alzheimer’s disease spectrum. *Brain* 139 547–562. 10.1093/brain/awv338 26586695 PMC4805086

[B58] Jung E. Osswald M. Blaes J. Wiestler B. Sahm F. Schmenger T. (2017). Tweety-Homolog 1 Drives Brain Colonization of Gliomas. *J. Neurosci.* 37 6837–6850. 10.1523/JNEUROSCI.3532-16.2017 28607172 PMC6705725

[B59] Jutten K. Mainz V. Delev D. Gauggel S. Binkofski F. Wiesmann M. (2020). Asymmetric tumor-related alterations of network-specific intrinsic functional connectivity in glioma patients. *Hum. Brain Mapp.* 41 4549–4561. 10.1002/hbm.25140 32716597 PMC7555062

[B60] Jutten K. Weninger L. Mainz V. Gauggel S. Binkofski F. Wiesmann M. (2021). Dissociation of structural and functional connectomic coherence in glioma patients. *Sci. Rep.* 11:16790. 10.1038/s41598-021-95932-5 34408195 PMC8373888

[B61] Kamoun A. Idbaih A. Dehais C. Elarouci N. Carpentier C. Letouze E. (2016). Integrated multi-omics analysis of oligodendroglial tumours identifies three subgroups of 1p/19q co-deleted gliomas. *Nat. Commun.* 7:11263. 10.1038/ncomms11263 27090007 PMC4838899

[B62] Kazuhiro K. Werner R. A. Toriumi F. Javadi M. S. Pomper M. G. Solnes L. B. (2018). Generative Adversarial Networks for the Creation of Realistic Artificial Brain Magnetic Resonance Images. *Tomography* 4 159–163. 10.18383/j.tom.2018.00042 30588501 PMC6299742

[B63] Kesler S. R. Harrison R. A. Petersen M. L. Rao V. Dyson H. Alfaro-Munoz K. (2019). Pre-surgical connectome features predict IDH status in diffuse gliomas. *Oncotarget* 10 6484–6493. 10.18632/oncotarget.27301 31741712 PMC6849657

[B64] Kesler S. R. Noll K. Cahill D. P. Rao G. Wefel J. S. (2017). The effect of IDH1 mutation on the structural connectome in malignant astrocytoma. *J. Neurooncol.* 131 565–574. 10.1007/s11060-016-2328-1 27848136 PMC5377918

[B65] Korgaonkar M. S. Goldstein-Piekarski A. N. Fornito A. Williams L. M. (2020). Intrinsic connectomes are a predictive biomarker of remission in major depressive disorder. *Mol. Psychiatry* 25 1537–1549. 10.1038/s41380-019-0574-2 31695168 PMC7303006

[B66] Lambin P. Rios-Velazquez E. Leijenaar R. Carvalho S. van Stiphout R. G. Granton P. (2012). Radiomics: extracting more information from medical images using advanced feature analysis. *Eur. J. Cancer* 48 441–446. 10.1016/j.ejca.2011.11.036 22257792 PMC4533986

[B67] Lee J. Wang N. Turk S. Mohammed S. Lobo R. Kim J. (2020). Discriminating pseudoprogression and true progression in diffuse infiltrating glioma using multi-parametric MRI data through deep learning. *Sci. Rep.* 10:20331. 10.1038/s41598-020-77389-0 33230285 PMC7683728

[B68] Li Y. Tang C. Lu J. Wu J. Chang E. F. (2021). Human cortical encoding of pitch in tonal and non-tonal languages. *Nat. Commun.* 12:1161. 10.1038/s41467-021-21430-x 33608548 PMC7896081

[B69] Liao X. Vasilakos A. V. He Y. (2017). Small-world human brain networks: perspectives and challenges. *Neurosci. Biobehav. Rev.* 77 286–300. 10.1016/j.neubiorev.2017.03.018 28389343

[B70] Liston C. Chen A. C. Zebley B. D. Drysdale A. T. Gordon R. Leuchter B. (2014). Default mode network mechanisms of transcranial magnetic stimulation in depression. *Biol. Psychiatry* 76 517–526. 10.1016/j.biopsych.2014.01.023 24629537 PMC4209727

[B71] Logothetis N. K. (2008). What we can do and what we cannot do with fMRI. *Nature* 453 869–878. 10.1038/nature06976 18548064

[B72] Logothetis N. K. Wandell B. A. (2004). Interpreting the BOLD signal. *Annu. Rev. Physiol.* 66 735–769. 10.1146/annurev.physiol.66.082602.092845 14977420

[B73] Lundervold A. S. Lundervold A. (2019). An overview of deep learning in medical imaging focusing on MRI. *Z. Med. Phys.* 29 102–127. 10.1016/j.zemedi.2018.11.002 30553609

[B74] Makin J. G. Moses D. A. Chang E. F. (2020). Machine translation of cortical activity to text with an encoder-decoder framework. *Nat. Neurosci.* 23 575–582. 10.1038/s41593-020-0608-8 32231340 PMC10560395

[B75] Mallela A. N. Peck K. K. Petrovich-Brennan N. M. Zhang Z. Lou W. Holodny A. I. (2016). Altered Resting-State Functional Connectivity in the Hand Motor Network in Glioma Patients. *Brain Connect.* 6 587–595. 10.1089/brain.2016.0432 27457676 PMC6913111

[B76] Malonek D. Grinvald A. (1996). Interactions between electrical activity and cortical microcirculation revealed by imaging spectroscopy: implications for functional brain mapping. *Science* 272 551–554. 10.1126/science.272.5261.551 8614805

[B77] Mandal A. S. Romero-Garcia R. Hart M. G. Suckling J. (2020). Genetic, cellular, and connectomic characterization of the brain regions commonly plagued by glioma. *Brain* 143 3294–3307. 10.1093/brain/awaa277 33278823 PMC7891236

[B78] Maniar Y. M. Peck K. K. Jenabi M. Gene M. Holodny A. I. (2021). Functional MRI Shows Altered Deactivation and a Corresponding Decrease in Functional Connectivity of the Default Mode Network in Patients with Gliomas. *Am. J. Neuroradiol.* 42 1505–1512. 10.3174/ajnr.A7138 33985945 PMC8367628

[B79] Martin-Subero M. Fuentes-Claramonte P. Salgado-Pineda P. Salavert J. Arevalo A. Bosque C. (2021). Autobiographical memory and default mode network function in schizophrenia: an fMRI study. *Psychol. Med.* 51 121–128. 10.1017/S0033291719003052 31680659 PMC7856411

[B80] Menon V. (2011). Large-scale brain networks and psychopathology: a unifying triple network model. *Trends Cogn. Sci.* 15 483–506. 10.1016/j.tics.2011.08.003 21908230

[B81] Misiura M. B. Howell J. C. Wu J. Qiu D. Parker M. W. Turner J. A. (2020). Race modifies default mode connectivity in Alzheimer’s disease. *Transl. Neurodegener.* 9:8. 10.1186/s40035-020-0186-4 32099645 PMC7029517

[B82] Morales H. (2021). Current and Future Challenges of Functional MRI and Diffusion Tractography in the Surgical Setting: from Eloquent Brain Mapping to Neural Plasticity. *Semin. Ultrasound CT MR* 42 474–489. 10.1053/j.sult.2021.07.005 34537116

[B83] Mori S. Barker P. B. (1999). Diffusion magnetic resonance imaging: its principle and applications. *Anat. Rec.* 257 102–109. 10.1002/(SICI)1097-0185(19990615)257:3<102::AID-AR7<3.0.CO;2-6 10397783

[B84] Mwansisya T. E. Hu A. Li Y. Chen X. Wu G. Huang X. (2017). Task and resting-state fMRI studies in first-episode schizophrenia: a systematic review. *Schizophr. Res.* 189 9–18. 10.1016/j.schres.2017.02.026 28268041

[B85] Naser M. A. Deen M. J. (2020). Brain tumor segmentation and grading of lower-grade glioma using deep learning in MRI images. *Comput. Biol. Med.* 121:103758. 10.1016/j.compbiomed.2020.103758 32568668

[B86] Niu C. Zhang M. Min Z. Rana N. Zhang Q. Liu X. (2014). Motor network plasticity and low-frequency oscillations abnormalities in patients with brain gliomas: a functional MRI study. *PLoS One* 9:e96850. 10.1371/journal.pone.0096850 24806463 PMC4013133

[B87] Ogawa S. Lee T. M. (1990). Magnetic resonance imaging of blood vessels at high fields: in vivo and in vitro measurements and image simulation. *Magn. Reson. Med.* 16 9–18. 10.1002/mrm.1910160103 2255240

[B88] Ogawa S. Tank D. W. Menon R. Ellermann J. M. Kim S. G. Merkle H. (1992). Intrinsic signal changes accompanying sensory stimulation: functional brain mapping with magnetic resonance imaging. *Proc. Natl. Acad. Sci. U. S. A.* 89 5951–5955. 10.1073/pnas.89.13.5951 1631079 PMC402116

[B89] Oh S. W. Harris J. A. Ng L. Winslow B. Cain N. Mihalas S. (2014). A mesoscale connectome of the mouse brain. *Nature* 508 207–214. 10.1038/nature13186 24695228 PMC5102064

[B90] Olszowy W. Aston J. Rua C. Williams G. B. (2019). Accurate autocorrelation modeling substantially improves fMRI reliability. *Nat. Commun.* 10:1220. 10.1038/s41467-019-09230-w 30899012 PMC6428826

[B91] Oosterhof N. N. Wiestler T. Downing P. E. Diedrichsen J. (2011). A comparison of volume-based and surface-based multi-voxel pattern analysis. *Neuroimage* 56 593–600. 10.1016/j.neuroimage.2010.04.270 20621701

[B92] Osswald M. Jung E. Sahm F. Solecki G. Venkataramani V. Blaes J. (2015). Brain tumour cells interconnect to a functional and resistant network. *Nature* 528 93–98. 10.1038/nature16071 26536111

[B93] Ostrom Q. T. Patil N. Cioffi G. Waite K. Kruchko C. Barnholtz-Sloan J. S. (2020). CBTRUS Statistical Report: primary Brain and Other Central Nervous System Tumors Diagnosed in the United States in 2013-2017. *Neuro Oncol.* 22 iv1–iv96. 10.1093/neuonc/noaa200 33123732 PMC7596247

[B94] Otten M. L. Mikell C. B. Youngerman B. E. Liston C. Sisti M. B. Bruce J. N. (2012). Motor deficits correlate with resting state motor network connectivity in patients with brain tumours. *Brain* 135 1017–1026. 10.1093/brain/aws041 22408270 PMC3326259

[B95] Park J. E. Kim H. S. Kim S. J. Kim J. H. Shim W. H. (2016). Alteration of long-distance functional connectivity and network topology in patients with supratentorial gliomas. *Neuroradiology* 58 311–320. 10.1007/s00234-015-1621-6 26635295

[B96] Qiu T. M. Gong F. Y. Gong X. Wu J. S. Lin C. P. Biswal B. B. (2017). Real-Time Motor Cortex Mapping for the Safe Resection of Glioma: an Intraoperative Resting-State fMRI Study. *Am. J. Neuroradiol.* 38 2146–2152. 10.3174/ajnr.A5369 28882861 PMC7963570

[B97] Raichle M. E. (2015). The brain’s default mode network. *Annu. Rev. Neurosci.* 38 433–447. 10.1146/annurev-neuro-071013-014030 25938726

[B98] Rudie J. D. Weiss D. A. Colby J. B. Rauschecker A. M. Laguna B. Braunstein S. (2021). Three-dimensional U-Net Convolutional Neural Network for Detection and Segmentation of Intracranial Metastases. *Radiol. Artif. Intell.* 3:e200204. 10.1148/ryai.2021200204 34136817 PMC8204134

[B99] Saad Z. S. Reynolds R. C. (2012). Suma. *Neuroimage* 62 768–773. 10.1016/j.neuroimage.2011.09.016 21945692 PMC3260385

[B100] Sarwar T. Tian Y. Yeo B. T. T. Ramamohanarao K. Zalesky A. (2021). Structure-function coupling in the human connectome: a machine learning approach. *Neuroimage* 226:117609. 10.1016/j.neuroimage.2020.117609 33271268

[B101] Satpute A. B. Lindquist K. A. (2019). The Default Mode Network’s Role in Discrete Emotion. *Trends Cogn. Sci.* 23 851–864. 10.1016/j.tics.2019.07.003 31427147 PMC7281778

[B102] Seeley W. W. (2019). The Salience Network: a Neural System for Perceiving and Responding to Homeostatic Demands. *J. Neurosci.* 39 9878–9882. 10.1523/JNEUROSCI.1138-17.2019 31676604 PMC6978945

[B103] Semmel E. S. Quadri T. R. King T. Z. (2021). Graph Theoretical Analysis of Brain Network Characteristics in Brain Tumor Patients: a Systematic Review. *Neuropsychol. Rev.* [Epub Online ahead of print] 10.1007/s11065-021-09512-5 34235627

[B104] Silva M. A. See A. P. Essayed W. I. Golby A. J. Tie Y. (2018). Challenges and techniques for presurgical brain mapping with functional MRI. *Neuroimage Clin.* 17 794–803. 10.1016/j.nicl.2017.12.008 29270359 PMC5735325

[B105] Sporns O. (2011). The human connectome: a complex network. *Ann. N. Y. Acad. Sci.* 1224 109–125. 10.1111/j.1749-6632.2010.05888.x 21251014

[B106] Sporns O. Tononi G. Kotter R. (2005). The human connectome: a structural description of the human brain. *PLoS Comput. Biol.* 1:e42. 10.1371/journal.pcbi.0010042 16201007 PMC1239902

[B107] Spreng R. N. Sepulcre J. Turner G. R. Stevens W. D. Schacter D. L. (2013). Intrinsic Architecture Underlying the Relations among the Default, Dorsal Attention, and Frontoparietal Control Networks of the Human Brain. *J. Cogn. Neurosci.* 25 74–86. 10.1162/jocn_a_00281 22905821 PMC3816715

[B108] Stam C. J. (2014). Modern network science of neurological disorders. *Nat. Rev. Neurosci.* 15 683–695. 10.1038/nrn3801 25186238

[B109] Stoecklein V. M. Stoecklein S. Galie F. Ren J. Schmutzer M. Unterrainer M. (2020). Resting-state fMRI detects alterations in whole brain connectivity related to tumor biology in glioma patients. *Neuro Oncol.* 22 1388–1398. 10.1093/neuonc/noaa044 32107555 PMC7523460

[B110] Sun W. Tang Y. Qiao Y. Ge X. Mather M. Ringman J. M. (2020). A probabilistic atlas of locus coeruleus pathways to transentorhinal cortex for connectome imaging in Alzheimer’s disease. *Neuroimage* 223:117301. 10.1016/j.neuroimage.2020.117301 32861791 PMC7797167

[B111] Tang F. Liang S. Zhong T. Huang X. Deng X. Zhang Y. (2020). Postoperative glioma segmentation in CT image using deep feature fusion model guided by multi-sequence MRIs. *Eur. Radiol.* 30 823–832. 10.1007/s00330-019-06441-z 31650265

[B112] Toga A. W. Clark K. A. Thompson P. M. Shattuck D. W. Van Horn J. D. (2012). Mapping the human connectome. *Neurosurgery* 71 1–5. 10.1227/NEU.0b013e318258e9ff 22705717 PMC3555558

[B113] Tomaszewski M. R. Gillies R. J. (2021). The Biological Meaning of Radiomic Features. *Radiology* 298 505–516. 10.1148/radiol.2021202553 33399513 PMC7924519

[B114] Turesky T. K. Vanderauwera J. Gaab N. (2021). Imaging the rapidly developing brain: current challenges for MRI studies in the first five years of life. *Dev. Cogn. Neurosci.* 47:100893. 10.1016/j.dcn.2020.100893 33341534 PMC7750693

[B115] Tzourio-Mazoyer N. Landeau B. Papathanassiou D. Crivello F. Etard O. Delcroix N. (2002). Automated anatomical labeling of activations in SPM using a macroscopic anatomical parcellation of the MNI MRI single-subject brain. *Neuroimage* 15 273–289. 10.1006/nimg.2001.0978 11771995

[B116] van den Heuvel M. P. Hulshoff Pol H. E. (2010). Exploring the brain network: a review on resting-state fMRI functional connectivity. *Eur. Neuropsychopharmacol.* 20 519–534. 10.1016/j.euroneuro.2010.03.008 20471808

[B117] van den Heuvel M. P. Sporns O. (2019). A cross-disorder connectome landscape of brain dysconnectivity. *Nat. Rev. Neurosci.* 20 435–446. 10.1038/s41583-019-0177-6 31127193 PMC8864539

[B118] van den Heuvel M. P. Kersbergen K. J. de Reus M. A. Keunen K. Kahn R. S. Groenendaal F. (2015). The Neonatal Connectome During Preterm Brain Development. *Cereb. Cortex* 25 3000–3013. 10.1093/cercor/bhu095 24833018 PMC4537441

[B119] Van Essen D. C. Smith S. M. Barch D. M. Behrens T. E. J. Yacoub E. Ugurbil K. (2013). The WU-Minn Human Connectome Project: an overview. *Neuroimage* 80 62–79. 10.1016/j.neuroimage.2013.05.041 23684880 PMC3724347

[B120] Van Essen D. C. Ugurbil K. Auerbach E. Barch D. Behrens T. E. J. Bucholz R. (2012). The Human Connectome Project: a data acquisition perspective. *Neuroimage* 62 2222–2231. 10.1016/j.neuroimage.2012.02.018 22366334 PMC3606888

[B121] Vassal M. Charroud C. Deverdun J. Le Bars E. Molino F. Bonnetblanc F. (2017). Recovery of functional connectivity of the sensorimotor network after surgery for diffuse low-grade gliomas involving the supplementary motor area. *J. Neurosurg.* 126 1181–1190. 10.3171/2016.4.JNS152484 27315027

[B122] Venkataramani V. Tanev D. I. Kuner T. Wick W. Winkler F. (2021). Synaptic input to brain tumors: clinical implications. *Neuro Oncol.* 23 23–33. 10.1093/neuonc/noaa158 32623467 PMC7850064

[B123] Venkataramani V. Tanev D. I. Strahle C. Studier-Fischer A. Fankhauser L. Kessler T. (2019). Glutamatergic synaptic input to glioma cells drives brain tumour progression. *Nature* 573 532–538. 10.1038/s41586-019-1564-x 31534219

[B124] Venkatesh H. S. Morishita W. Geraghty A. C. Silverbush D. Gillespie S. M. Arzt M. (2019). Electrical and synaptic integration of glioma into neural circuits. *Nature* 573 539–545. 10.1038/s41586-019-1563-y 31534222 PMC7038898

[B125] Wang Y. Du H. Xia M. Ren L. Xu M. Xie T. (2013). A hybrid CPU-GPU accelerated framework for fast mapping of high-resolution human brain connectome. *PLoS One* 8:e62789. 10.1371/journal.pone.0062789 23675425 PMC3651094

[B126] Warmuth C. Gunther M. Zimmer C. (2003). Quantification of blood flow in brain tumors: comparison of arterial spin labeling and dynamic susceptibility-weighted contrast-enhanced MR imaging. *Radiology* 228 523–532. 10.1148/radiol.2282020409 12819338

[B127] Watts D. J. Strogatz S. H. (1998). Collective dynamics of ‘small-world’ networks. *Nature* 393 440–442. 10.1038/30918 9623998

[B128] Wei Y. Li C. Cui Z. Zou J. Wong A. L. K. C. Sinha R. (2021). Structural connectome quantifies tumor invasion and predicts survival in glioblastoma patients. *bioRxiv* [Preprint]. 10.1101/2021.03.09.434656 PMC10115235 36189936

[B129] Weil S. Osswald M. Solecki G. Grosch J. Jung E. Lemke D. (2017). Tumor microtubes convey resistance to surgical lesions and chemotherapy in gliomas. *Neuro Oncol.* 19 1316–1326. 10.1093/neuonc/nox070 28419303 PMC5596180

[B130] Wen P. Y. Packer R. J. (2021). The 2021 WHO Classification of Tumors of the Central Nervous System: clinical implications. *Neuro Oncol.* 23 1215–1217. 10.1093/neuonc/noab120 34185090 PMC8328017

[B131] Xu H. Ding S. Hu X. Yang K. Xiao C. Zou Y. (2013). Reduced efficiency of functional brain network underlying intellectual decline in patients with low-grade glioma. *Neurosci. Lett.* 543 27–31. 10.1016/j.neulet.2013.02.062 23562503

[B132] Yan J. Zhao Y. Chen Y. Wang W. Duan W. Wang L. (2021). Deep learning features from diffusion tensor imaging improve glioma stratification and identify risk groups with distinct molecular pathway activities. *EBioMedicine* 72:103583. 10.1016/j.ebiom.2021.103583 34563923 PMC8479635

[B133] Yang J. Gohel S. Zhang Z. Hatzoglou V. Holodny A. I. Vachha B. A. (2021). Glioma-Induced Disruption of Resting-State Functional Connectivity and Amplitude of Low-Frequency Fluctuations in the Salience Network. *Am. J. Neuroradiol.* 42 551–558. 10.3174/ajnr.A6929 33384293 PMC7959416

[B134] Yeshurun Y. Nguyen M. Hasson U. (2021). The default mode network: where the idiosyncratic self meets the shared social world. *Nat. Rev. Neurosci.* 22 181–192. 10.1038/s41583-020-00420-w 33483717 PMC7959111

[B135] Yu Z. Tao L. Qian Z. Wu J. Liu H. Yu Y. (2016). Altered brain anatomical networks and disturbed connection density in brain tumor patients revealed by diffusion tensor tractography. *Int. J. Comput. Assist. Radiol. Surg.* 11 2007–2019. 10.1007/s11548-015-1330-y 26914530

[B136] Yuan B. Zhang N. Yan J. Cheng J. Lu J. Wu J. (2020). Tumor grade-related language and control network reorganization in patients with left cerebral glioma. *Cortex* 129 141–157. 10.1016/j.cortex.2020.04.015 32473401

[B137] Yuan T. Zuo Z. Ying J. Jin L. Kang J. Gui S. (2020). Structural and Functional Alterations in the Contralesional Medial Temporal Lobe in Glioma Patients. *Front. Neurosci.* 14:10. 10.3389/fnins.2020.00010 32153348 PMC7044242

[B138] Zeng D. Ye Z. Shen R. Yu G. Wu J. Xiong Y. (2021). IOBR: multi-Omics Immuno-Oncology Biological Research to Decode Tumor Microenvironment and Signatures. *Front. Immunol.* 12:687975. 10.3389/fimmu.2021.687975 34276676 PMC8283787

[B139] Zhang H. Ille S. Sogerer L. Schwendner M. Schroder A. Meyer B. (2021). Elucidating the structural-functional connectome of language in glioma-induced aphasia using nTMS and DTI. *Hum. Brain Mapp.* [Epub Online ahead of print] 10.1002/hbm.25757 34951084 PMC8933329

[B140] Zhang L. Giuste F. Vizcarra J. C. Li X. Gutman D. (2020). Radiomics Features Predict CIC Mutation Status in Lower Grade Glioma. *Front. Oncol.* 10:937. 10.3389/fonc.2020.00937 32676453 PMC7333647

[B141] Zhao Z. Zhang K. N. Wang Q. Li G. Zeng F. Zhang Y. (2021). Chinese Glioma Genome Atlas (CGGA): a Comprehensive Resource with Functional Genomic Data from Chinese Glioma Patients. *Genom. Proteom. Bioinform.* 19 1–12. 10.1016/j.gpb.2020.10.005 33662628 PMC8498921

[B142] Zhu D. Yuan T. Gao J. Xu Q. Xue K. Zhu W. (2021). Correlation between cortical gene expression and resting-state functional network centrality in healthy young adults. *Hum. Brain Mapp.* 42 2236–2249. 10.1002/hbm.25362 33570215 PMC8046072

